# The collateral map: prediction of lesion growth and penumbra after acute anterior circulation ischemic stroke

**DOI:** 10.1007/s00330-023-10084-6

**Published:** 2023-08-30

**Authors:** Jin Seok Yi, Hee Jong Ki, Yoo Sung Jeon, Jeong Jin Park, Taek-Jun Lee, Jin Tae Kwak, Sang Bong Lee, Hyung Jin Lee, In Seong Kim, Joo Hyun Kim, Ji Sung Lee, Hong Gee Roh, Hyun Jeong Kim

**Affiliations:** 1 Department of Neurosurgery, Daejeon St. Mary’s Hospital, College of Medicine, The Catholic University of Korea, Daejeon, Republic of Korea; 2 Department of Neurosurgery, Konkuk University Medical Center, Konkuk University School of Medicine, Seoul, Republic of Korea; 3 Department of Neurology, Konkuk University Medical Center, Konkuk University School of Medicine, Seoul, Republic of Korea; 4 Department of Neurosurgery, Kangwon National University College of Medicine, Chuncheon, Republic of Korea; 5 Department of Neurology, Daejeon St. Mary’s Hospital, College of Medicine, The Catholic University of Korea, Daejeon, Republic of Korea; 6 School of Electrical Engineering, Korea University, Seoul, Republic of Korea; 7 Siemens Healthineers Ltd., Seoul, Republic of Korea; 8 Philips Healthcare Korea, Seoul, Republic of Korea; 9 Clinical Research Center, Asan Institute for Life Science, Asan Medical Center, University of Ulsan College of Medicine, Seoul, Republic of Korea; 10 Department of Radiology, Konkuk University Medical Center, Konkuk University School of Medicine, 120-1 Neungdong-Ro, Kwangjin-Gu, Seoul, 05030 Republic of Korea; 11 Department of Radiology, Daejeon St. Mary’s Hospital, College of Medicine, The Catholic University of Korea, 64 Daeheung-Ro, Jung-Gu, Daejeon, 34943 Republic of Korea

**Keywords:** Cerebrovascular disorders, Stroke, Collateral circulation, Magnetic resonance imaging, Magnetic resonance angiography

## Abstract

**Objectives:**

This study evaluated the collateral map’s ability to predict lesion growth and penumbra after acute anterior circulation ischemic strokes.

**Methods:**

This was a retrospective analysis of selected data from a prospectively collected database. The lesion growth ratio was the ratio of the follow-up lesion volume to the baseline lesion volume on diffusion-weighted imaging (DWI). The time-to-maximum (Tmax)/DWI ratio was the ratio of the baseline Tmax  > 6 s volume to the baseline lesion volume. The collateral ratio was the ratio of the hypoperfused lesion volume of the phase_FU (phase with the hypoperfused lesions most approximate to the follow-up DWI lesion) to the hypoperfused lesion volume of the phase_baseline of the collateral map. Multiple logistic regression analyses were conducted to identify independent predictors of lesion growth. The concordance correlation coefficients of Tmax/DWI ratio and collateral ratio for lesion growth ratio were analyzed.

**Results:**

Fifty-two patients, including twenty-six males (mean age, 74 years), were included. Intermediate (OR, 1234.5; *p* < 0.001) and poor collateral perfusion grades (OR, 664.7; *p* = 0.006) were independently associated with lesion growth. Phase_FUs were immediately preceded phases of the phase_baselines in intermediate or poor collateral perfusion grades. The concordance correlation coefficients of the Tmax/DWI ratio and collateral ratio for the lesion growth ratio were 0.28 (95% CI, 0.17–0.38) and 0.88 (95% CI, 0.82–0.92), respectively.

**Conclusion:**

Precise prediction of lesion growth and penumbra can be possible using collateral maps, allowing for personalized application of recanalization treatments. Further studies are needed to generalize the findings of this study.

**Clinical relevance statement:**

Precise prediction of lesion growth and penumbra can be possible using collateral maps, allowing for personalized application of recanalization treatments.

**Key Points:**

*• Cell viability in cerebral ischemia due to proximal arterial steno-occlusion mainly depends on the collateral circulation.*

*• The collateral map shows salvageable brain extent, which can survive by recanalization treatments after acute anterior circulation ischemic stroke.*

*• Precise estimation of salvageable brain makes it possible to make patient-specific treatment decision.*

**Supplementary Information:**

The online version contains supplementary material available at 10.1007/s00330-023-10084-6.

## Introduction

The primary goal of recanalization treatments is to save the salvageable brain (penumbra) to improve functional outcomes. Recently, the HERMES data showed that the follow-up infarct volume was a strong independent predictor of functional outcome [1]. Accurate information of the penumbral extent can be the most direct indicator for selecting patients for treatments. Currently, the penumbra is estimated with a threshold of  > 6 s of the contralateral time-to-maximum (Tmax > 6 s) on CT or MR perfusion imaging [2]. However, comparative studies using perfusion imaging and positron emission tomography have shown that perfusion imaging tends to overestimate the volume of the penumbra [3]. This can lead to the inclusion of patients who are unlikely to benefit from recanalization treatments, as they may have only a minimal amount of salvageable brain. The optimal time-to-maximum (Tmax) threshold for delineating the penumbra is still a matter of controversy [4], and the results for calculating Tmax can vary depending on the software used, even with the same threshold [5, 6]. These factors may have an impact on patient selection for treatments.

Cell viability and functional outcomes in cerebral ischemia due to proximal arterial steno-occlusion mainly depend on the collateral circulation, which varies among patients. Previous studies have shown that good collaterals slow down infarct growth and poor collaterals accelerate it. Therefore, better collaterals are associated with less infarct growth and better functional outcome, whereas poor collaterals are linked to hemorrhagic complications and unfavorable functional outcome even in successful recanalization [7–10]. However, the current collateral imaging methods, such as multiphase CT angiography, the prominent vessel sign on susceptibility-weighted imaging, or arterial transit artifact on arterial spin labeling, provide only peripheral vessel information without incorporating the underlying tissue status in a dynamic dimension. Therefore, these methods have limitations in regional estimations of the penumbra [11–13]. We aimed to evaluate the ability of collateral circulation imaging, named the “collateral map,” to predict lesion growth and penumbra in patients with acute ischemic stroke due to large vessel steno-occlusion in the anterior circulation.

## Materials and methods

The local institutional review boards of Konkuk Medical Center and Daejeon St. Mary’s Hospital approved this study, and written informed consent was obtained from all participants.

### Patients

For this retrospective analysis, we selected patients from the data in the ongoing Database of Acute ischemic Stroke Analysis Network (DASAN) that contains clinical and imaging data of patients with acute ischemic stroke due to large vessel occlusion, and the data were prospectively collected from two university hospitals from January 1, 2016. The inclusion criteria of DASAN are as follows: (1) participants older than 18 years of age, (2) participants with acute ischemic stroke due to occlusion or severe stenosis of the internal carotid artery and/or M1 or M2 segment of the middle cerebral artery, or the basilar artery, and (3) participants who underwent brain CT and MR imaging including diffusion-weighted imaging (DWI), susceptibility-weighted imaging, dynamic contrast-enhanced MR angiography, dynamic susceptibility MR perfusion, and fluid-attenuated inversion recovery at admission. The inclusion criteria for this study were as follows: (1) patients with steno-occlusion of the internal carotid artery and/or M1 or M2 segment of the middle cerebral artery (MCA) who were evaluated within 8 h of symptom onset, (2) patients with follow-up DWI and angiography within 7 days, and (3) patients who presented with unchanged steno-occlusive arterial lesions on follow-up angiography. Patients with premorbid modified Rankin scale scores greater than 2, hemorrhagic transformation, procedure-related complications such as thromboembolism or subarachnoid hemorrhage, and patients who underwent craniectomy were excluded. The patients were evaluated based on demographic data, medical history, vascular risk factors, routine blood tests, brain imaging, and cardiological tests. The severity of stroke was assessed with the National Institutes of Health Stroke Scale (NIHSS). Functional outcomes were assessed on day 90 with the modified Rankin scale; a favorable functional outcome was defined as a modified Rankin scale score of 2 or less on day 90.

### Imaging protocol, postprocessing, and analysis

MRI imaging was performed with 3-Tesla MRI scanners (Magnetom Skyra, Siemens Healthineers and Ingenia, Philips Healthcare). The acquisition parameters were the same as those used in a previous study [14]. A neurologist (T.J.L. with 16 years of experience) who was blinded to all the clinical and other imaging data measured the baseline lesion volume and follow-up lesion volume on DWI by manual drawing using Medical Image Processing, Analysis, and Visualization (MIPAV; version 7.1.1; National Institutes of Health). The lesion growth ratio was the ratio of the follow-up lesion volume to the baseline lesion volume, which represented penumbral extent. Lesion growth was defined as a lesion growth ratio  ≥ 1.2, considering the impact of vasogenic edema on the follow-up lesion volume. The baseline Tmax  > 6 s volume was automatically calculated with RAPID software (RAPID; iSchemaView). The Tmax/DWI ratio was the ratio of the baseline Tmax  > 6 s volume to the baseline lesion volume.

**Fig. 2:**
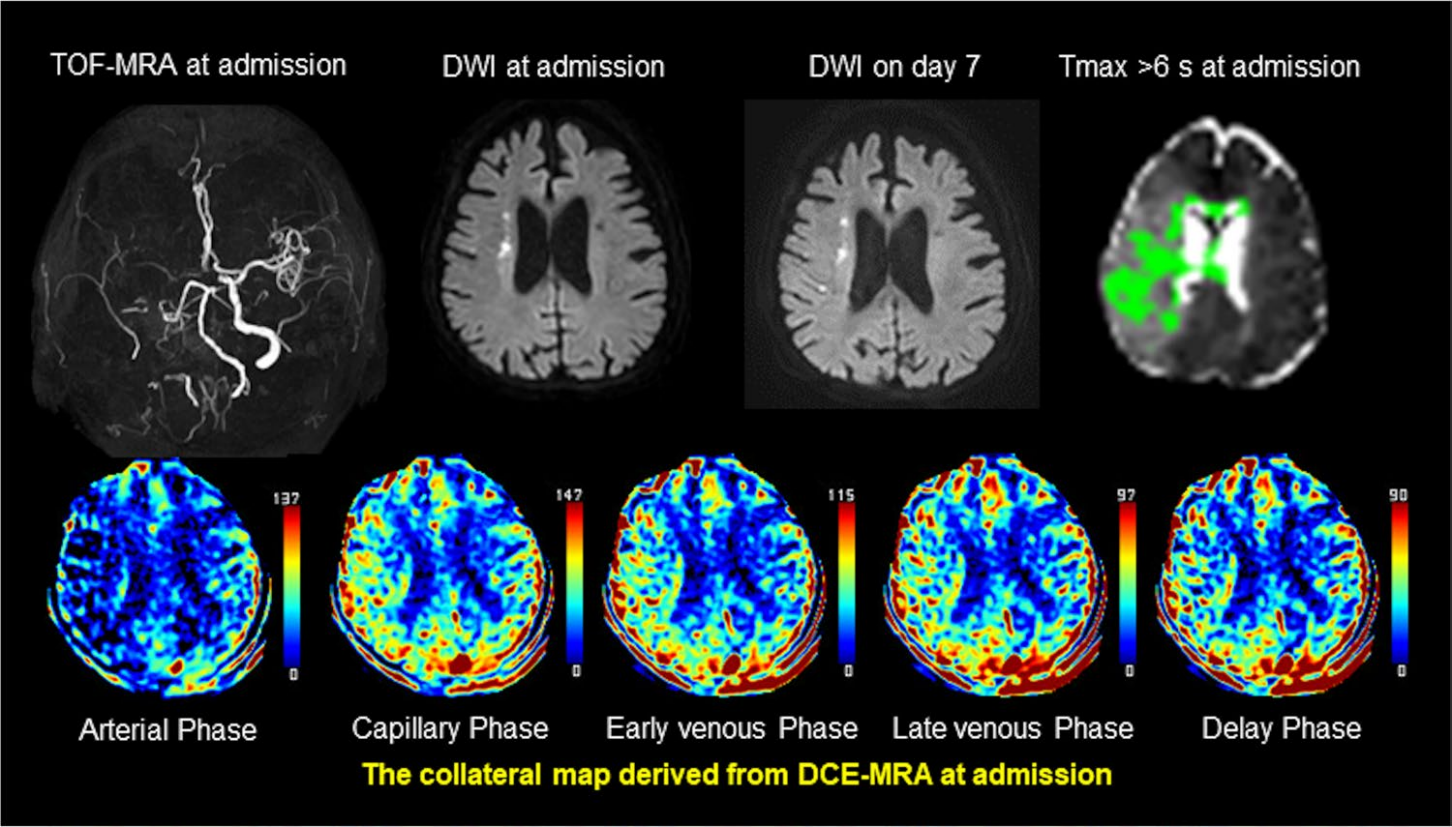
Case of good collateral perfusion grade. Images of an elderly patient with occlusion of the right internal carotid and middle cerebral arteries demonstrated on time-of-flight MR angiography (TOF-MRA). The premorbid modified Rankin scale score of this patient was 0, and the National Institutes of Health Stroke Scale score at admission was 5. The patient underwent intravenous thrombolysis followed by intraarterial thrombectomy, but recanalization of the occluded arteries was not achieved. Diffusion-weighted imaging (DWI) at admission showed acute infarction in the right middle cerebral artery territory. The collateral map derived from dynamic contrast-enhanced magnetic resonance angiography (DCE-MRA) at admission shows a good collateral perfusion status (MR acute ischemic stroke collateral perfusion score of 5: no collateral perfusion delay in the middle cerebral artery territory in the capillary phase). The baseline DWI lesion did not grow significantly on DWI on day 7. The phase_baseline and phase_FU cannot be determined in the collateral map because there is no hypoperfused lesion approximate to the baseline and follow-up DWI lesions on the collateral map. An MR perfusion image with a threshold of  > 6 s of the contralateral time-to-maximum (Tmax > 6 s) shows a large penumbra (green area). Tmax > 6 s overestimates the final infarct extent

With dynamic contrast-enhanced MR angiography source data, we generated collateral maps by using an in-house program, which was composed of images in the arterial, capillary, early venous, late venous, and delay phases (Figs. 1 and 2). The phases of the collateral map were automatically divided based on arterial and venous signal intensity-time curves obtained from ROIs on the normal side MCA and the superior sagittal sinus. The details of the methodology used were the same as those used in previous studies [14, 15].

**Fig.**
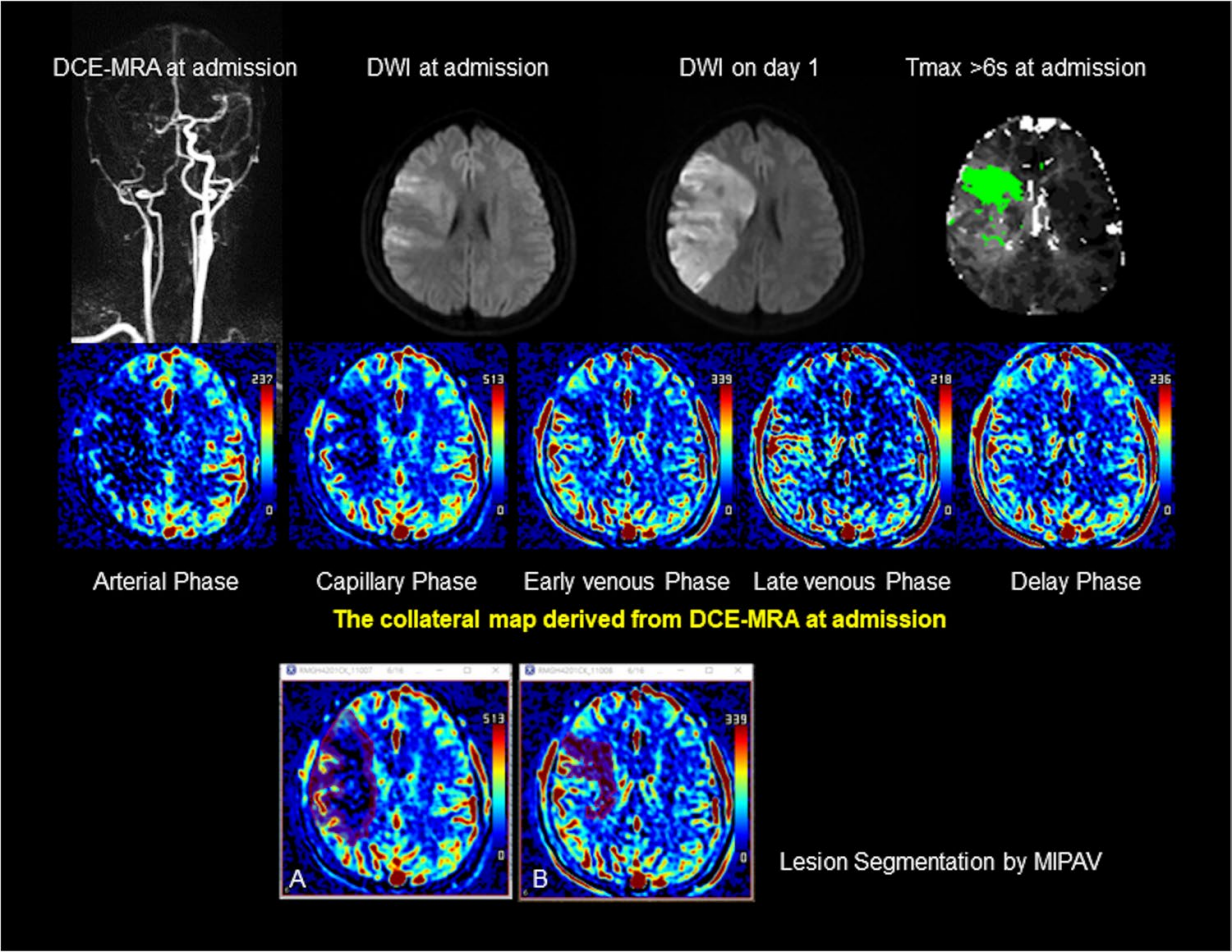
Case of intermediate collateral perfusion grade. Images of a middle-aged patient with occlusion of the right internal carotid and middle cerebral arteries demonstrated on dynamic contrast-enhanced MR angiography (DCE-MRA). The premorbid modified Rankin scale score of this patient was 0, and the National Institutes of Health Stroke Scale score at admission was 11. The patient underwent intravenous thrombolysis followed by intraarterial thrombectomy, but recanalization of the occluded arteries was not achieved. Diffusion-weighted imaging (DWI) at admission showed an acute infarction in the right middle cerebral artery territory. The collateral map derived from DCE-MRA at admission shows an intermediate collateral perfusion status (MR acute ischemic stroke collateral perfusion score of 2: collateral perfusion delay more than one-half of the middle cerebral artery territory in the capillary phase and equal to or less than one-half in the early venous phase). The DWI lesion extent at admission coincides with the hypoperfused lesion on the early venous phase of the collateral map, so the early venous phase is determined as the baseline lesion phase. DWI on day 1 shows that the lesion growth covers the entire hypoperfused lesion seen on the capillary phase of the collateral map at admission, so the capillary phase is determined as the phase_FU. A and B are images displayed on Medical Image Processing, Analysis, and Visualization (MIPAV; version 7.1.1; National Institutes of Health), showing the hypoperfused lesion painted on images in the capillary and early venous phases, respectively, for measuring the volume of the hypoperfused lesion of the collateral map. The precise collateral ratio was calculated as the ratio of the hypoperfused lesion volume in the phase_FU to the hypoperfused lesion volume in the phase_baseline. The lesion (green area) with a threshold of  > 6 s of the contralateral time-to-maximum (Tmax > 6 s) on MR perfusion imaging is similar to the baseline DWI lesion. The collateral map shows the extents of baseline lesion and final infarction precisely, but Tmax  > 6 s underestimates the final infarct extent

Two raters (H.G.R. with 20 years of experience and H.J.K. with 7 years of experience as a neuroradiologist and a neurosurgeon, respectively) who were blinded to all the clinical and other imaging data independently graded the collateral perfusion status of the collateral map using the collateral perfusion scores as follows: 5, excellent; 4, good; 3, intermediate to good; 2, intermediate to poor; 1, poor; and 0, very poor (Table 1) [14, 15]. Two raters determined the final collateral perfusion scores by consensus. A neurologist (S.B.L. with 22 years of experience) and a neurosurgeon (H.J.L. with 20 years of experience) who were blinded to all the clinical and other imaging data except DWI independently determined the phase_baseline and phase_FU of the collateral map by visual estimation on separate occasions 1 week apart. The phase_baseline and phase_FU were determined as phases with the hypoperfused lesions most approximate to the baseline and follow-up DWI lesions, respectively (Fig. 1). Two raters determined the final phases by consensus. A neuroradiologist (H.J.K. with 18 years of experience) and a neurosurgeon (Y.S.J. with 6 years of experience), who were blinded to all the clinical and other imaging data, measured the hypoperfused lesion volume in the capillary phases, phase_baselines and phase_FUs, using MIPAV software as follows. In the capillary phase, the areas with less perfusion in the ischemic hemisphere compared to the perfused contralateral hemisphere and, in other phases, the persisting regions of hypoperfused lesion from the capillary phase, were manually painted with free drawing brush tool on each slice (Fig. 1A and B). MIPAV software automatically calculated the painted area volumes of all slices by converting each painted area on each slice to a voxel of interest (VOI). The final hypoperfused lesion volume was determined by the average value of the volumes measured by two measurers. The collateral ratio (CR) was measured by two methods. Precise CR (pCR) was the ratio of the hypoperfused lesion volume of the phase_FU to the hypoperfused lesion volume of the phase_baseline, and approximate CR (aCR) was the ratio of the hypoperfused lesion volume of the capillary phase to the hypoperfused lesion volume of the early venous phase.

**Table 1:** Collateral perfusion grading system for analysis of the collateral map

Collateral perfusion score	Description of collateral perfusion status
5 (excellent)	No or small* collateral perfusion delay† in the ischemic MCA territory in the capillary phase regardless of the collateral perfusion status in the arterial phase
4 (good)	Collateral perfusion delay ≤ ½ of the ischemic MCA territory in the capillary phase AND no or small collateral perfusion delay in the early venous phases
3 (intermediate to good))	1) Collateral perfusion delay ≤ ½ of the ischemic MCA territory in the capillary and early venous phases 2) Collateral perfusion delay > ½ of the ischemic MCA territory in the capillary phase AND no or small collateral perfusion delay in the early venous phase
2 (intermediate to poor)	Collateral perfusion delay > ½ of the ischemic MCA territory in the capillary phase AND ≤ ½ of the ischemic MCA territory in the early venous phase
1 (poor)	Collateral perfusion delay > ½ of the ischemic MCA territory in the early venous phase AND ≤ ½ of the ischemic MCA territory in the late venous phase
0 (very poor)	Collateral perfusion delay > ½ of the ischemic MCA territory in the late venous phase

*MCA*, middle cerebral artery

^*^ “Small” indicates an area smaller than 1 out of 8 MCA regions, which are divided as follows: the insula, subcortical structures (basal ganglia and internal capsule), and M1–M6 regions of the Alberta Stroke Programme Early CT Scores (ASPECTS). In visually undetermined, borderline cases with a collateral perfusion delay of approximately one-half of the MCA territory, the collateral perfusion score was decided by counting the number of regions with a collateral perfusion delay. For example, a collateral perfusion delay greater than one-half of the MCA territory means that the collateral perfusion delay involves  > 4 of the 8 regions in the MCA territory

^†^In the capillary phase, collateral perfusion delay in the ischemic MCA territory is evaluated in comparison to the perfusion of the contralateral MCA territory. In the early venous, late venous, and delay phases, the region that consistently demonstrates collateral perfusion delay observed in the capillary phase is regarded as collateral perfusion delay of the ischemic MCA territory

### Statistical analysis

Statistical analysis was performed using SAS (SAS, version 9.4; Institute Inc.). The patient characteristics were expressed as the mean (SD), median [interquartile range (IQR)], or number of patients (%). We reclassified the collateral perfusion grading as follows: good collateral perfusion = collateral perfusion scores 5 and 4, intermediate collateral perfusion = collateral perfusion scores 3 and 2, and poor collateral perfusion = collateral perfusion scores 1 and 0 considering the small study population. Differences in the distribution of the patient characteristics among the collateral perfusion grades were identified using the chi-square test, Fisher’s exact test, ANOVA, and Kruskal‒Wallis test, as appropriate. The interrater reliabilities for collateral perfusion grading, determination of phase_baseline and phase_FU, and measurement of the hypoperfused lesion volume of the collateral map were measured by the Cohen weighted κ. Univariate logistic regression analysis was performed to identify independent predictors of lesion growth. Candidate predictors were presented in Table 2. The significant predictors with a *p* value of  < 0.05 in the univariate analysis were included in the multiple logistic regression analysis with Firth’s correction [16, 17]. The results of logistic regression are reported as odds ratios (ORs) with 95% confidence intervals (CIs). The AUC, sensitivity, specificity, accuracy, positive predictive value, and negative predictive value were constructed to evaluate the prediction performance of Tmax/DWI ratio and precise and approximate CRs for lesion growth. Comparisons of performance in the prediction of lesion growth between CR and Tmax/DWI ratio were made using the Delong test, McNemar’s test, and generalized score statistics according to the type of variable. Concordance correlation coefficients were used to measure agreement between Tmax/DWI ratio (or CRs) and lesion growth ratio. The difference in concordance correlation coefficients was analyzed by the bias-corrected and accelerated bootstrap method. The results are reported as odds ratios (ORs) with 95% confidence intervals (CIs). *p* < 0.05 was considered to indicate statistical significance.

**Table 2:** Patient characteristics (*n* = 52) according to the collateral perfusion grades based on the collateral map

Characteristics	Good	Intermediate	Poor	*p* value
No. of patients	16 (30.8)	29 (55.8)	7 (13.5)	
Men	10 (62.5)	14 (48.3)	2 (28.6)	0.34
Age (y) *	74 ± 8	73 ± 14	78 ± 8	0.69
Risk factors				
Hypertension	11 (68.8)	19 (65.5)	3 (42.9)	0.56
Diabetes	4 (25.0)	6 (20.7)	1 (14.3)	0.90
Hyperlipidemia	5 (31.3)	6 (20.7)	2 (28.6)	0.67
Atrial fibrillation	1 (6.3)	13 (44.8)	3 (42.9)	0.01
Current smoker	1 (6.3)	4 (13.8)	0 (0.0)	0.67
Daily alcohol consumption	0 (0.0)	2 (6.9)	0 (0.0)	0.65
Previous transient ischemic attack	0 (0.0)	0 (0.0)	1 (14.3)	0.13
Previous stroke	3 (18.8)	4 (13.8)	2 (28.6)	0.60
Previous ischemic heart disease	3 (18.8)	8 (27.6)	2 (28.6)	0.82
Family history of stroke	0 (0.0)	3 (10.3)	0 (0.0)	0.71
Baseline NIHSS score†	2 (2–5)	8 (3–14)	14 (10–17)	< 0.001
Baseline DWI lesion volume (mL) †	3.1 (1.3–3.8)	11.8 (4.4–26.7)	59.7 (5.5–65.5)	0.001
Follow-up DWI lesion volume (mL) †	3.1 (1.5–4.0)	33.2 (21.6–65.6)	187.9 (158.8–244.4)	< 0.001
‡Lesion growth ratio†	1.1 (1.0–1.1)	2.8 (2.1–3.8)	3.1 (2.4–30.4)	< 0.001
^§^Functional outcome				< 0.001
Favorable	13 (86.7)	6 (26.1)	0 (0.0)	
Unfavorable	2 (13.3)	17 (73.9)	4 (100.0)	

The mean overall patient age was 74 years ± 12 (standard deviation) (26 men and 26 women). Unless otherwise noted, the data are presented as the number of patients, with percentage in parentheses

*DWI*, diffusion-weighted imaging; *NIHSS*, National Institutes of Health Stroke Scale

^*^Data are means ± standard deviations

^†^Data are presented as the medians, with interquartile ranges in parentheses

^‡^Lesion growth ratio is the ratio of the follow-up DWI lesion volume to the baseline DWI lesion volume

^§^Favorable functional outcome was defined as a modified Rankin scale score less than or equal to 2, and unfavorable functional outcome was defined as a score greater than 2 at day 90

## Results

### Patient characteristics

**Fig. 3:**
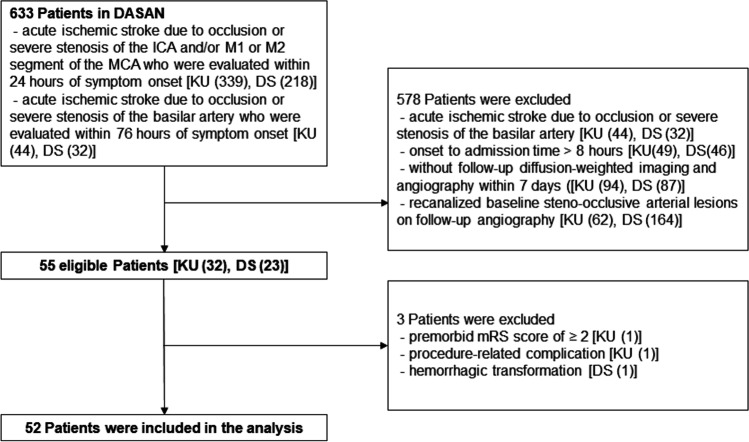
Flowchart shows the patient enrollment. The numbers in parentheses represent the number of patients at Konkuk University Medical Center (KU) and Daejeon St. Mary;s Hospital (DS). DASAN, Database of Acute ischemic Stroke Analysis Network, which is a database of ongoing prospective observational study of acute ischemic stroke due to large vessel occlusion from two university hospitals since 2016; mRS, modified Rankin scale

Of the 633 patients in DASAN between January 1, 2016, and March 3, 2021, 52 patients were included in the current analysis (Fig. 3). The mean age was 74 ± 12 years (range = 33–90 years), 50% were men (26 men and 26 women), and the median baseline NIHSS was 6 (IQR 2–13). The median imaging follow-up interval was 2 days (range 1–7 days, IQR 1–6). The demographic findings among the patients with different collateral perfusion grades are presented in Table 2. The presence of atrial fibrillation (*p* = 0.015), a higher NIHSS score, larger baseline lesion volume and follow-up lesion volume, a higher lesion growth ratio, and unfavorable functional outcomes were associated with a worse collateral perfusion grade (*p* ≤ 0.001).

### Imaging analysis for DWI, Tmax > 6 s, and the collateral map

The median baseline lesion volume and follow-up lesion volume were 6.0 mL (range: 0.6–118.9 mL; IQR: 2.5, 21.6) and 22.5 mL (range: 0.8–246.2 mL; IQR: 4.3, 70.8 mL), respectively. The median lesion growth ratio was 5.8 (range: 1–52; IQR: 1, 4). Thirty-six patients (69.2%) showed lesion growth. The median Tmax > 6 s lesion volume and Tmax/DWI ratio were 67 mL (range: 0–282 mL; IQR: 14, 105 mL) and 16.6 (range: 0–196.4; IQR: 1.5, 10.5), respectively. The interrater reliability was almost perfect for collateral perfusion grading of the collateral map (weighted *κ* = 0.98; 95% CI: 0.95–1.00) and for determination of the phase_baseline (weighted *κ* = 1.00; 95% CI: 1.00–1.00) and phase_FU (weighted *κ* = 0.91; 95% CI: 0.82–1.00). A good collateral perfusion grade was obtained in 16 patients (30.8%), an intermediate collateral perfusion grade was obtained in 29 (55.8%), and a poor collateral perfusion grade was obtained in 7 (13.5%). Of the 16 patients with a good collateral perfusion grade, the phase_baseline and phase_FU were undetermined in 14 patients because there were no hypoperfused lesions of the collateral maps approximate to such small DWI lesions (Table 2) (Fig. 2). The phase_baseline and phase_FU were the same in the remaining 2 patients. One was the early venous phase, and the other was the capillary phase. There were no patients with lesion growth. The phase_baseline and phase_FU of the 36 participants with an intermediate or a poor collateral perfusion grade are presented in Table 3. The phase_FUs were immediately preceded phases of the phase_baselines. In the phase_baseline, all 28 participants with early venous phase showed capillary phase in the phase_FU, while all 7 participants with late venous phase showed early venous phase in the phase_FU. The interrater reliability was almost perfect (weighted *κ* = 0.99; 95% CI: 0.98–0.99) for the measurement of the hypoperfusion lesion volume of the collateral map. The median pCR was 2.5 (range: 1.0–34.1; IQR: 1.0, 3.8), and the median aCR was 2.4 (range: 1.0–34.1; IQR: 1.0, 4.4).

**Table 3:** Phase_baseline* and Phase_FU^†^ of patients with an intermediate or a poor collateral perfusion grade (*n* = 36)

	Phase_baseline		
	Early venous phase	Late venous phase	Delay phase
	*n* = 28 (77.8%)	*n* = 7 (19.4%)	*n* = 1 (2.8%)
Phase_FU	Capillary phase	Early venous phase	Late venous phase

^*^Phase_baseline is a phase of the collateral map with the hypoperfused lesions most approximate to the baseline diffusion-weighted imaging lesion

^†^Phase_FU is a phase of the collateral map with the hypoperfused lesions most approximate to the follow-up diffusion-weighted imaging lesion

### Multiple logistic regression analyses to identify predictive variables for lesion growth

In the univariable analysis, the presence of atrial fibrillation (OR, 12.0; 95% CI: 1.43–100.79; *p* = 0.02), higher baseline NIHSS (OR, 1.38; 95% CI: 1.11–1.71; *p* = 0.004), intermediate collateral perfusion grade (OR, 1946.9; 95% CI: 33.5–113,012.3; *p* < 0.001), and poor collateral perfusion grade (OR, 494.99; 95% CI: 7.3–33,550.4; *p* = 0.004) were associated with lesion growth. In the multivariable analysis, intermediate collateral perfusion grade (OR, 1234.5; 95% CI: 20.0–76,048.3; *p* < 0.001) and poor collateral perfusion grade (OR, 664.7; 95% CI: 6.4–68,811.8; *p* = 0.006) were independently associated with lesion growth. However, the results of the multivariable analysis were unstable because none of the patients had lesion growth within the group of patients with good collateral perfusion grades. Unfavorable functional outcomes were associated with lesion growth in the univariable analysis (OR, 22.8; 95% CI: 4.0–130.1; *p* < 0.001).

### Performance of Tmax/DWI Ratio, aCR, and pCR for the prediction of lesion growth and lesion growth ratio

The AUC, sensitivity, specificity, accuracy, positive predictive value, and negative predictive value of the Tmax/DWI ratio, pCR, and aCR for lesion growth are presented in Table 4. The AUC, specificity, accuracy, and positive predictive value of the precise and approximate CR for lesion growth were significantly higher than those of the Tmax/DWI ratio (*p* < 0.05).

**Table 4:** Performance of the Tmax/DWI ratio*, approximate collateral ratio (CR)^†^, and precise CR‡ for prediction of lesion growth^§^

	AUC	Sensitivity	Specificity	Accuracy	PPV	NPV
Tmax/DWI ratio	0.8 (0.6–0.9)	94.4 (81.3–99.3)	62.5 (35.4–84.8)	84.6 (71.9–93.1)	85.0 (70.2–94.3)	83.3 (51.6–97.9)
Approximate CR	1.0 (1.0–1.0)	97.2 (85.5–99.9)	100.0 (79.4–100.0)	98.1 (89.7–100.0)	100.0 (90.0–100.0)	94.1 (71.3–99.9)
Precise CR	1.0 (1.0–1.0)	97.2 (85.5–99.9)	100.0 (79.4–100.0)	98.1 (89.7–100.0)	100.0 (90.0–100.0)	94.1 (71.3–99.9)
*p* value^∥^	0.005	0.56	0.01	0.04	0.008	0.37

Unless otherwise noted, the data are described as the performance with 95% confidence intervals in parentheses

*AUC* area under the receiver operating characteristic curve, *DWI* diffusion-weighted imaging, *NPV* negative predictive value, *PPV* positive predictive value, *Tmax* time-to-maximum

^*^Tmax/DWI ratio was the ratio of the lesion volume with  > 6 s delay of the contralateral time-to-maximum of magnetic resonance perfusion imaging to the baseline DWI lesion volume

^†^Approximate CR was the ratio of the hypoperfused lesion volume of the capillary phase to the hypoperfused lesion volume of the early venous phase of the collateral map

^‡^Precise CR was the ratio of the hypoperfused lesion volume of the phase_FU to the hypoperfused lesion volume of the phase_basline of the collateral map. The phase_baseline and phase_FU were determined as phases with the hypoperfused lesions most approximate to the baseline and follow-up DWI lesions, respectively

^§^Lesion growth was defined as a  ≥ 1.2 lesion growth ratio of the follow-up DWI lesion volume to the baseline DWI lesion volume

^∥^*p* value for comparing performance in the prediction of lesion growth between CR and the Tmax/DWI ratio using Delong test, McNemar’s test, and generalized score statistics according to the type of variable. The performance of the approximate and precise CR was the same

**Fig. 4:**
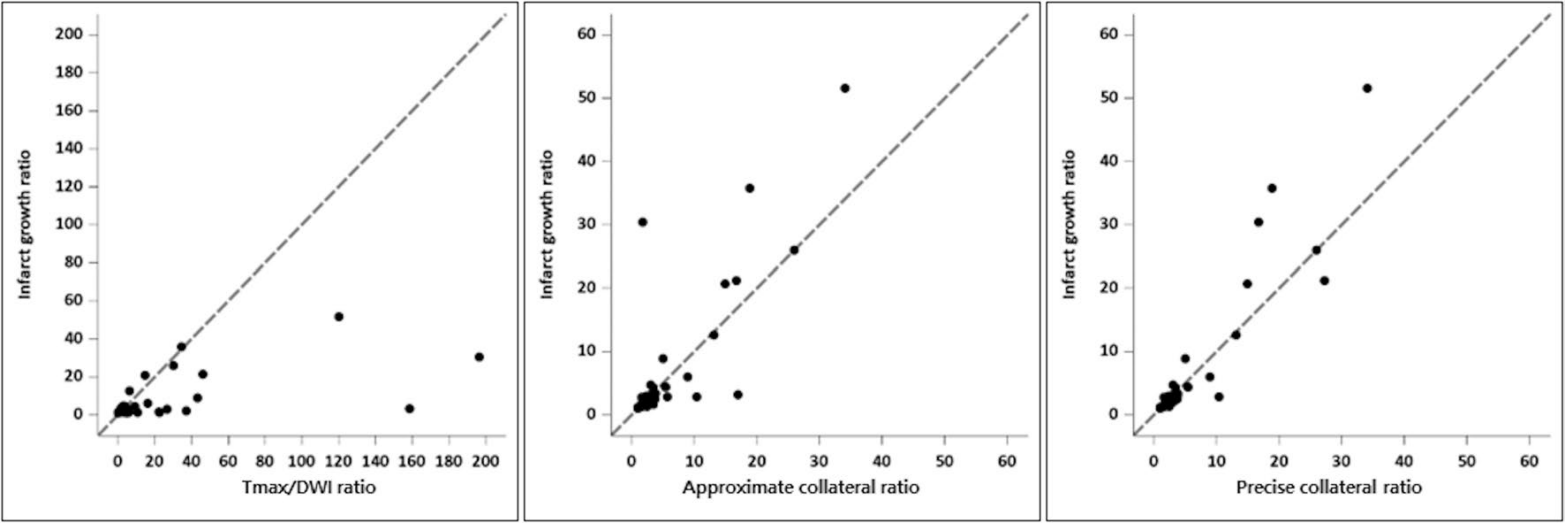
Scatterplot for the concordance correlation coefficient of the time-to maximum (Tmax)/diffusion-weighted imaging (DWI) ratio, approximate collateral ratio, and precise collateral ratio with the lesion growth ratio. Scatter plots show the superiority of precise and approximate collateral ratios of the collateral map in correlation with the lesion growth ratio, which represents penumbral extent

The concordance correlation coefficients of the Tmax/DWI ratio, aCR, and pCR for the lesion growth ratio were 0.28 (95% CI, 0.17–0.38), 0.77 (95% CI, 0.66–0.84), and 0.88 (95% CI, 0.82–0.92), respectively. The difference in the concordance correlation coefficient between the aCR and Tmax/DWI ratio was 0.49 (95% CI, 0.29–0.82); between the pCR and Tmax/DWI ratio, it was 0.60 (95% CI, 0.29–0.90); and between the pCR and aCR, it was 0.11 (95% CI, 0.003–0.53) (Fig. 4). Because the 95% CIs of differences in concordance correlation coefficients did not contain 0, there were statistically significant differences.

## Discussion

The main findings of this study for the fate of the baseline DWI lesion in patients with acute ischemic stroke due to steno-occlusion of the internal carotid artery and/or M1 or M2 segment of the MCA who were evaluated within 8 h of symptom onset are as follows: (1) the baseline DWI lesions with a good collateral perfusion status did not grow, (2) the baseline DWI lesions with an intermediate or a poor collateral perfusion status grew within the hypoperfused lesion that appeared on the phase of the collateral map that immediately preceded the one that shows the baseline lesion, and (3) the maximal range of lesion growth was the hypoperfused lesion on the capillary phase of the collateral map.

Regional assessment of the penumbra is conceptually all that may be required for acute decision making. However, the current method using perfusion imaging to estimate the penumbra is not accurate [18–20]. The current study also showed the significant inferiority of the Tmax > 6/DWI ratio compared with CR of the collateral map in the prediction of lesion growth and lesion growth ratio. The main cause of the inaccuracy is that perfusion imaging is a calculated snapshot based on a fixed time threshold that cannot represent the complex spatial and temporal ischemic process according to the individual hemodynamics [21]. Only a few studies have been reported for the prediction of tissue outcomes using collateral circulation imaging. Nannoni’s study using single-phase CT angiography for collateral estimation showed that better collaterals were associated with lower core volume but not with higher penumbra volumes [22]. The arterial scoring method based on single-phase CT angiography had a critical limitation of underestimating leptomeningeal collaterals with a longer transit time due to early triggering of a static acquisition [23]. Multiphase CT angiography overcame this limitation by 3 consecutive acquisitions after administration of contrast material to capture the delayed flow [24]. D′Esterre et al [25] reported that, by pattern analysis of washout and delayed filling of the pial collateral vessels, multiphase CT angiography showed similar performance to CT perfusion parameters in penumbral estimation. Multiphase CT angiography also has innate disadvantages for the regional assessment of the penumbra in that the phases are determined not by the patient’s hemodynamics but by the moving speed of the CT equipment, and it does not provide parenchymal perfusion status [26]. Therefore, collateral and regional penumbral estimation by multiphase CT angiography can be limited.

The collateral map derived from dynamic contrast-enhanced MR angiography or dynamic susceptibility contrast-enhanced MR perfusion is composed of the images of arterial, capillary, early venous, late venous, and delay phases, which are divided according to the patient’s hemodynamic status [14]. We can intuitively see when and how much collateral circulation comes in the ischemic brain, including the parenchymal perfusion status. This study was initiated from our observation that baseline or follow-up DWI lesions matched a hypoperfused lesion on a certain phase image of the collateral map and showed that the phase_FUs were immediately preceded phases of the phase_baselines (Fig. 1). We can revise the definition of pCR as the ratio of the hypoperfused lesion volume of the immediately preceding phase of the phase_baseline to the hypoperfused lesion volume of the phase_baseline. We also estimated the aCR because the baseline DWI lesion might be uncertain, and the follow-up DWI lesion was matched with a hypoperfused lesion of the capillary phase in nearly 78% of the patients with intermediate or poor collateral circulation. The current study showed the superiority of both CRs of the collateral map over the Tmax/DWI ratio in the prediction of penumbral extent represented by the lesion growth ratio. The best thing is that we can recognize the salvageable brain extent by simple visual estimation practically (Figs. 1 and 2). With knowledge of the future infarct extent, we can make different treatment decisions in octogenarian patients who are unlikely to benefit from risky treatments (Fig. 2) [27, 28]. We can accurately select patients who can still benefit from recanalization to prevent significant lesion growth regardless of the time window, even though they have mild symptoms or large baseline lesions that meet the exclusion criteria in the current guidelines [29–31]. A 3-min MR imaging protocol composed of DWI and dynamic contrast-enhanced MR angiography that provides accurate information on the baseline lesion, causative vessel, penumbra, and collateral perfusion status is possible in acute ischemic stroke. And if a CT collateral map capable of providing information similar to the results of this study is available, it would enhance the ability of CT evaluation in acute ischemic stroke (supplemental figure online).

The main limitation of this study is the small study population. It is very difficult to collect patients who are suitable for the inclusion criteria of this study, especially in the early time window, when most patients are eligible for recanalization treatments and successful recanalization is achieved in most patients. We could select only 52 patients among 633 patients in the prospectively collected cohort for 5 years and 3 months. The current study demonstrated the potential for precise prediction of the penumbra with the strict inclusion criteria and clear results. However, it may not account for the variable nature of the ischemic tissue across all six collateral-perfusion grades. Another limitation is the retrospective nature of the study. We performed all measures and assessments independently by two blinded investigators to minimize retrospective study bias. Further prospective studies in large populations are necessary to evaluate the value of CR for determining eligibility for recanalization treatments.

## Conclusions

Individual-based predictions of lesion growth and salvageable brain extent can be possible using the collateral map in patients with acute anterior circulation ischemic stroke. Precise prediction of the final infarct extent may enable patient-specific application of recanalization treatments and improve the results of the treatments. However, the results of this study, which are based on retrospective design and small sample size, should be interpreted with caution. Further prospective studies in large population from multiple centers are needed to confirm the reliability and generalizability of the findings.

## Supplfementary Information

Below is the link to the electronic supplementary material. Supplementary file1 (PDF 191 KB)

## Abbreviations

aCR: Approximate collateral ratio
DASAN: Database of Acute ischemic Stroke Analysis Network
DWI: Diffusion-weighted imaging
MCA: Middle cerebral artery
pCR: Precise collateral ratio
Tmax: Time-to-maximum

## Declarations

### Guarantor

The scientific guarantor of this publication in Hyun Jeong Kim.

### Statistics and biometry

One of the authors, Ji Sung Lee who is a statistics expert, conducted statistical analysis of this study and reviewed this manuscript.

### Study subjects or cohorts overlap

Twenty-five patients in this study subjects have been previously reported in the following 4 reports:

Roh HG, Kim EY, Kim IS et al (2019) A novel collateral imaging method derived from time-resolved dynamic contrast-enhanced mr angiography in acute ischemic stroke: a pilot study. AJNR Am J Neuroradiol 40:946-953

Kim HJ, Lee SB, Choi JW et al (2020) Multiphase MR angiography collateral map: functional outcome after acute anterior circulation ischemic stroke. Radiology 295:192-201

Lee HJ, Roh HG, Lee SB et al (2021) Collateral estimation by susceptibility-weighted imaging and prediction of functional outcomes after acute anterior circulation ischemic stroke. Sci Rep 11:21370

Lee TJ, Roh HG, Kim JH et al (2021) Collateral and permeability imaging derived from dynamic contrast material-enhanced MR angiography in prediction of PH 2 hemorrhagic transformation after acute ischemic stroke: a pilot study. Neuroradiology 63:1471-1479

The prior articles dealt with the predictability of the collateral map or susceptibility-weighted imaging for functional outcomes or hemorrhagic transformation of the patients with acute ischemic stroke whereas in this manuscript we report on the lesion growth and penumbra.

### Methodology

• Retrospective

• Observational

• Multicenter study based on an ongoing database of prospectively collected patients with acute ischemic stroke from two university hospitals
