# Supplementary material for: The collateral map: prediction of lesion growth and penumbra after acute anterior circulation ischemic stroke
Source: Eur Radiol. 2023 Aug 30;34(3):1411–21. doi: 10.1007/s00330-023-10084-6 (PMC10873223; doi:10.1007/s00330-023-10084-6)
Supplement: Supplementary file 1 — Supplementary file1 (PDF 191 KB) [file 330_2023_10084_MOESM1_ESM.pdf]

The Collateral Map: Prediction of Lesion Growth and Penumbra after Acute Anterior Circulation Ischemic Strokes

ELECTRONIC SUPPLEMENTARY MATERIAL

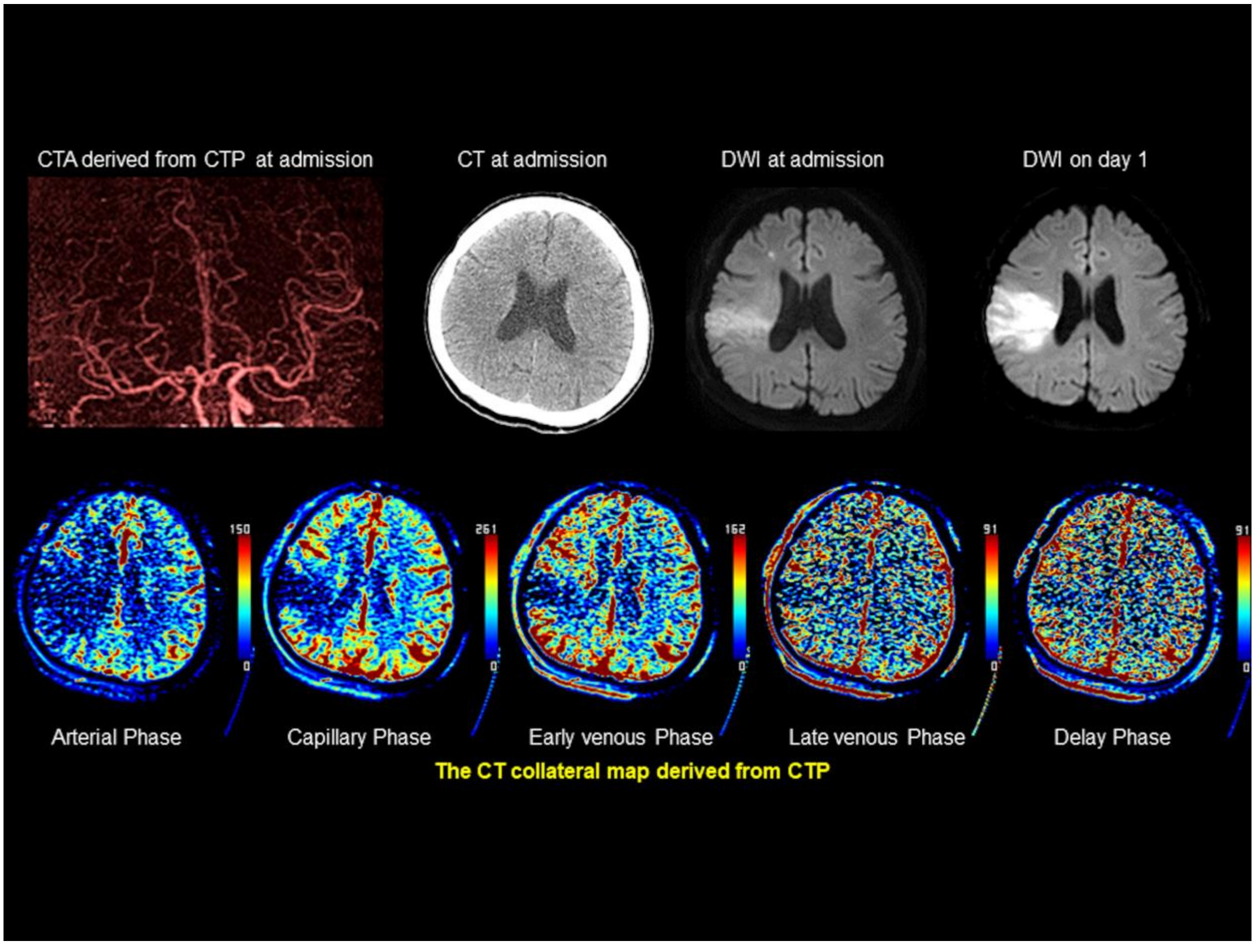

Supplemental Figure: Preliminary case of the computed tomography collateral map.

Images of a middle-aged patient with occlusion of the right internal carotid artery demonstrated on CT angiography (CTA) derived from the CT perfusion (CTP). The premorbid modified Rankin scale score of this patient was 0, and the National Institutes of Health Stroke Scale score at admission was 6. The patient underwent intravenous thrombolysis followed by intraarterial

thrombectomy, but recanalization of the occluded arteries was not achieved. At admission, brain CT image demonstrates uncertain differentiation between cortical gray matter and white matter, as well as sulcal effacement in the right frontoparietal region. However, determining the exact extent of the baseline infarct lesion is challenging. Diffusion-weighted imaging (DWI) obtained immediately after CT scan reveals acute infarct signals in the right middle cerebral artery territory. The CT collateral map derived from the CTP at admission shows an intermediate collateral perfusion status (collateral perfusion score of 3: collateral perfusion delay less than one-half of the middle cerebral artery territory in the capillary and early venous phase) that could predict infarct growth. The extent of the DWI lesion at admission closely matched the extent of the hypoperfused lesion observed on the early venous phase of the CT collateral map. On day 1, DWI shows that the baseline lesion expanded to the similar size as the hypoperfused lesion observed on the capillary phase of the CT collateral map at admission. Even without DWI, it may be possible to determine the baseline lesion and infarct growth using the CT collateral map. Additionally, by utilizing the approximate collateral ratio of this study, it may be possible to predict how much the baseline lesion will grow.
